# DNA methylation of *AHCY* may increase the risk of ischemic stroke

**DOI:** 10.17305/bjbms.2020.4535

**Published:** 2020-11

**Authors:** Lei Zhao, Xiaosheng Chen, Shengjun Zhou, Zhiqing Lin, Xi Yu, Yi Huang

**Affiliations:** 1 Department of Neurosurgery, Ningbo First Hospital, Ningbo, China; 2 Ningbo Hospital, Zhejiang University School of Medicine, Ningbo, China; 3 Key Laboratory, Ningbo Medical Center Lihuili Hospital, Ningbo China

**Keywords:** Ischemic stroke, DNA methylation, S-adenosylhomocysteine hydrolase, sex, age

## Abstract

Genetic factors play an important role in the pathogenesis of ischemic stroke. Of these, epigenetic modifications provide a new direction for the study of ischemic stroke pathogenesis. This study aimed to determine the correlation between DNA methylation of the gene encoding S-adenosylhomocysteine hydrolase (*AHCY*) and the risk of ischemic stroke in 64 ischemic stroke patients and 138 patients with traumatic brain injury (control group). The methylation level of *AHCY* was analyzed using quantitative methylation-specific polymerase chain reaction. Statistically significant differences in *AHCY* methylation levels were observed between the case group [medians (interquartile range): 0.13% (0.09%, 0.27%)] and the control group [0.06% (0.00%, 0.17%), *p* < 0.0001], and these associations remained significant in both male (*p* = 0.003) and female (*p* = 0.0005) subjects. A subgroup analysis by age revealed a considerably higher percentage of methylated *AHCY* in the case group than the control group in all age groups (age < 60 years, *p* = 0.007; age ≥ 60 years, *p* < 0.0001). A receiver operating characteristic (ROC) curve analysis revealed a trend toward a role for *AHCY* methylation as an indicator of risk in all ischemic patients [area under the curve (AUC) = 0.70, *p* = 0.0001], male patients (AUC = 0.67, *p* = 0.004), and female patients (AUC = 0.75, *p* = 0.0002). Our study confirmed a significant association between the *AHCY* DNA methylation level and the risk of ischemic stroke, suggesting that this gene methylation pattern may be a potential diagnostic marker of ischemic stroke.

## INTRODUCTION

Stroke is among the three leading causes of disease-related mortality in humans and a common cause of chronic disability in adults [1], and accordingly poses a serious risk to human health and places a heavy burden on society [2]. Stroke can be divided etiologically as ischemic or hemorrhagic stroke [3]. Regarding the former type, common risk factors such as diabetes, hypertension, and hyperlipidemia can explain only a small fraction of the actual risk [4]. Increasingly, studies have shown that genetic factors play key roles in the pathogenesis of ischemic stroke [5,6]. For example, genome-wide association studies have identified several genetic variations that contribute to the risk factors associated with ischemic stroke [7,8]. Other recent studies have indicated that epigenetic changes play important roles in stroke development [9,10], and even that the gap between environmental factors and ischemic stroke may be bridged by an epigenetic mechanism [11].

DNA methylation is an epigenetic mechanism by which the spatial structure of a DNA molecule is transformed to affect the interactions between DNA and proteins. Potentially, DNA hypermethylation could induce the silencing of genes and the loss of gene function [12]. Aberrant DNA methylation has been observed in various vascular developmental diseases, including brain arteriovenous malformations [13], intracranial aneurysms [14], and cerebral infarctions [15]. Furthermore, the DNA methylation level was reported as a key regulator of vascular smooth muscle cell dedifferentiation and vascular remodeling [16].

S-adenosylhomocysteine hydrolase (*AHCY*) is the only enzyme known to catalyze the hydrolysis of S-adenosyl-L-homocysteine (SAH) to yield homocysteine and adenosine. Hence, this enzyme can relieve the inhibitory activities of SAH on S-adenosyl methionine (SAM)-dependent transmethylation reactions [17,18]. However, dysfunctional *AHCY* activity can result in serious pathological consequences, such as childhood death [19], Alzheimer’s disease [20], Parkinson’s disease [21], age-related diseases [22], neuroblastoma [23], and large-artery atherosclerotic stroke [24]. Despite these negative outcomes, few studies have investigated the relationship between *AHCY* and the risk of ischemic stroke. As the homocysteine level is considered an independent predictor of severe neurological impairment in ischemic stroke patients, we hypothesized that DNA methylation of *AHCY* may play a key role in the pathological development of ischemic stroke. In this case-control study, we explored the role of DNA methylation of *AHCY* as a risk factor for ischemic stroke in a Chinese population.

## MATERIALS AND METHODS

### Samples and clinical data

Approval of the study procedure was provided by the Ethics Committee of the Ningbo First Hospital. The investigation included 64 ischemic stroke patients and 138 controls (patients with a traumatic brain injury without any vascular lesions) that visited the Stroke Center, Ningbo First Hospital, between September 2013 and December 2015. In all patients, the fulfillment of the diagnostic criteria based on international standardized definitions was confirmed using magnetic resonance imaging and cranial computed tomography scans. Any subject presenting with a serious liver or kidney disease or cardiovascular disease was excluded from the study.

Peripheral venous blood was collected from all patients in the morning after fasting and stored in tubes containing EDTA (anticoagulant). General information, including sex, age, and the levels of triglycerides (TG), total cholesterol (TC), high-density lipoprotein (HDL), low-density lipoprotein (LDL), apolipoprotein A (ApoA), apolipoprotein B (ApoB), and apolipoprotein E (ApoE), was collected for all patients. Bioindicators were measured using an automatic biochemical analyzer (AU2700; Olympus Corp., Tokyo, Japan).

### SYBR Green-based quantitative methylation-specific polymerase chain reaction (qMSP)

Genomic DNA was extracted from peripheral blood using the QIAamp DNA Mini Kit (Qiagen, Hilden, Germany). The DNA bisulfite conversion was assessed using the EZ DNA Methylation-Gold™ Kit (Zymo Research, Orange, CA, USA). The qMSP assay was performed using a LightCycler480 device (Roche Diagnostics, Mannheim, Germany). Human *ACTB* was used as the internal reference gene to standardize the amount of target DNA. The percentage of methylated reference (PMR) of the genes in each sample was calculated using the 2^−ΔΔCt^ quantification approach [25]. The sequences of specific primers were as follows: *AHCY* forward, 5´-GGTCGTAATCGGTTGAT-3´ and reverse, 5´-CAATTCCTATTCCCAAACTAAA-3´; *ACTB* forward, 5´-TGGTGATGGAGGAGGTTTAGTAAGT-3´ and reverse, 5´-AACCAATAAAACCTACTCCTCCCTTAA-3´. The entire protocol for qMSP was previously described [26].

### Statistical analysis

Statistical data were analyzed and plotted using SPSS Version 20.0 (IBM Corp, Armonk, NY, USA) and GraphPad Prism version 8.0 (GraphPad Software, La Jolla, CA, USA). Measured data were assessed using the one-sample Kolmogorov–Smirnov test for normality, and normally distributed data were assessed using the independent samples *t*-test. The data are presented as means ± standard deviations. Nonparametric tests were used to assess data that did not conform to a normal distribution, and these data are expressed as medians (interquartile range). A receiver operating characteristic (ROC) curve analysis was performed to evaluate the sensitivity of *AHCY* DNA methylation as an indicator of ischemic stroke diagnosis. All statistical tests were bilateral. Statistical significance was defined as a *p* value < 0.05.

## RESULTS

The clinical characteristics of patients in the ischemic stroke and control groups are shown in Table 1. The HDL level was considerably lower in stroke patients (1.03 ± 0.27) than in controls (1.13 ± 0.26, *p* = 0.022). No significant differences were observed with respect to the other demographic and clinical characteristics (age, sex, hypertension, diabetes, alcohol consumption, smoking, TG, TC, LDL, ApoA, ApoB, and ApoE; *p* > 0.05) between the two groups.

**TABLE 1:**
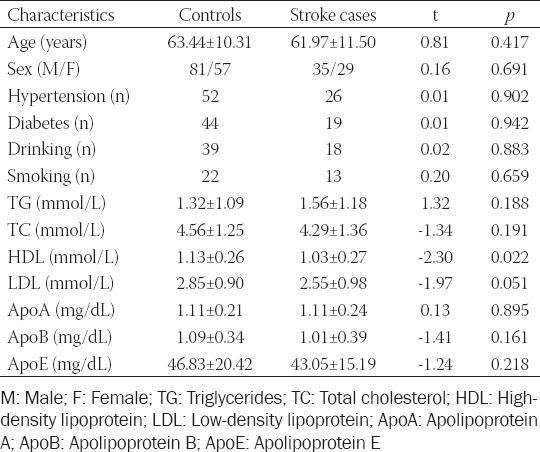
Clinical characteristics of all participants

**FIGURE 1:**
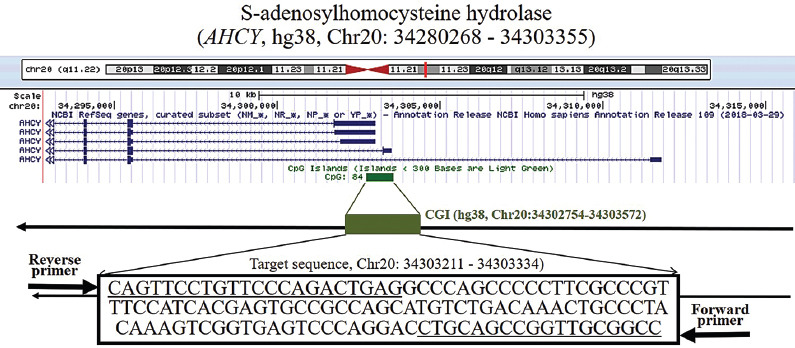
Characteristics of target sequences in S-adenosylhomocysteine hydrolase (AHCY)

**FIGURE 2:**
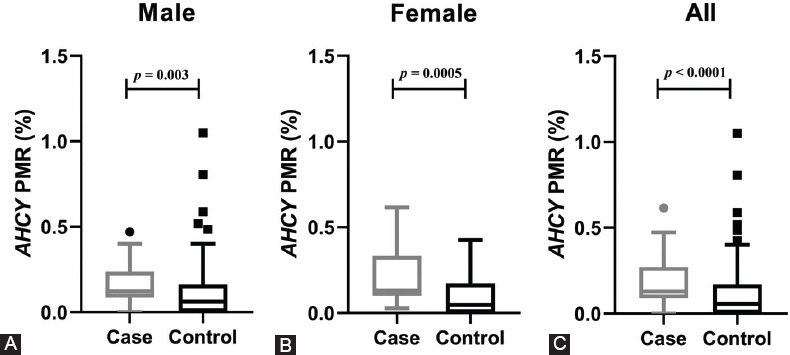
Comparison of S-adenosylhomocysteine hydrolase (*AHCY*) methylation levels between cases and controls in a sex-stratified subgroup analysis. (A) The percentage of methylated reference (PMR) levels of *AHCY* in male ischemic cases [0.12% (0.09%, 0.24%)] were higher than those in male controls [0.06% (0.00%, 0.16%), *p* = 0.003]. (B) The PMR levels of *AHCY* in female ischemic cases [0.13% (0.10%, 0.33%)] were higher than those in female controls [0.05% (0.00%, 0.17%), *p* = 0.0005]. (C) The PMR level of *AHCY* in all ischemic stroke cases [0.13% (0.09%, 0.27%)] was significantly higher than in the controls [0.06% (0.00%, 0.17%), *p* < 0.0001].

**FIGURE 3:**
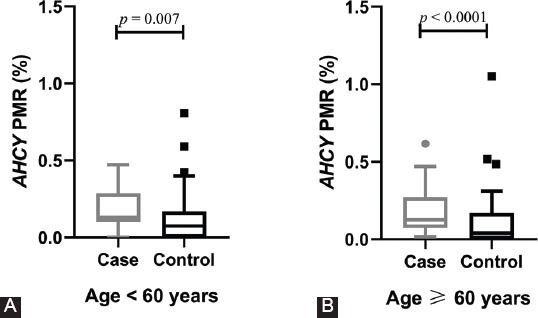
Comparison of S-adenosylhomocysteine hydrolase (*AHCY*) methylation levels in a subgroup analysis stratified by age. (A) In the <60 years age group, the percentage of methylated reference (PMR) levels of *AHCY* were higher in the ischemic cases than in the controls [0.13% (0.10%, 0.29) vs. 0.07% (0.00%, 0.17%), *p* = 0.007]. (B) In the ≥60 years age group, the PMR levels of *AHCY* were higher in the ischemic cases than that in the controls [0.13% (0.07%, 0.27%) vs. 0.04% (0.00%, 0.17%), *p* < 0.0001].

Target sequences (Chr20: 34303211–34303334) located in the CGI region of *AHCY* were selected to determine the DNA methylation level (Figure 1). As shown in Figure 2, the PMR level of *AHCY* was significantly higher in ischemic stroke patients [0.13% (0.09%, 0.27%)] than in controls [0.06% (0.00%, 0.17%), *p* < 0.0001]. A subgroup analysis stratified by sex similarly demonstrated significantly higher PMR levels of *AHCY* in both male [0.12% (0.09%, 0.24%)] and female ischemic stroke patients [0.13% (0.10%, 0.33%)] relative to controls [0.06% (0.00%, 0.16%), *p* = 0.003 and 0.05% (0.00%, 0.17%), *p* = 0.0005, respectively]. A subgroup analysis stratified by age similarly revealed a significantly higher PMR level of *AHCY* in the ischemic stroke patients relative to the controls, regardless of age group [<60 years, 0.13% (0.10%, 0.29) vs. 0.07% (0.00%, 0.17%), *p* = 0.007; age ≥60 years, 0.13% (0.07%, 0.27%) vs. 0.04% (0.00%, 0.17%), *p* < 0.0001; Figure 3).

**FIGURE 4:**
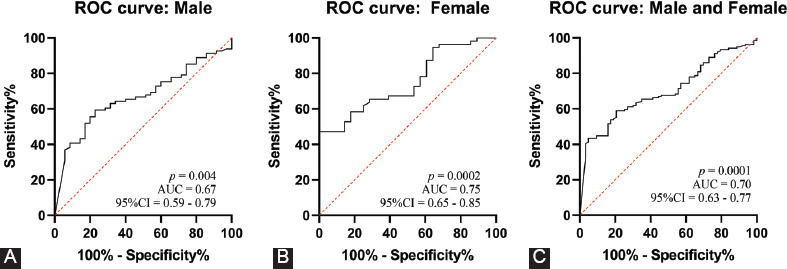
Receiver operating characteristic (ROC) curve analysis of the association between S-adenosylhomocysteine hydrolase (*AHCY*) DNA methylation and ischemic stroke. (A) In male patients, *AHCY* methylation tended to indicate a diagnosis of ischemic stroke [area under curve (AUC) = 0.67, 95% confidence interval (CI) = 0.59–0.79, *p* = 0.004]. (B) In female patients, *AHCY* methylation also tended to indicate a diagnosis of ischemic stroke (AUC = 0.75, 95% CI = 0.65–0.85, *p* = 0.0002). (C) In all patients, *AHCY* methylation tended to indicate a diagnosis of ischemic stroke (AUC = 0.70, 95% CI = 0.63–0.77, *p* = 0.0001).

As shown in Figure 4, a ROC analysis demonstrated a trend toward *AHCY* methylation as an indicator of the ischemic stroke risk in all ischemic patients [area under the curve (AUC) = 0.70, *p* = 0.0001), as well as in male (AUC = 0.67, *p* = 0.004) and female patients (AUC = 0.75, *p* = 0.0002).

## DISCUSSION

The development of the field of epigenetics has elicited significant interest in the correlations between human diseases and epigenetic factors. Particularly, epigenetic modifications provide a new direction for studies of the pathogenesis of ischemic stroke. Although epigenetic changes can result in heritable changes in gene expression, the underlying DNA sequence is not altered. Rather, these modifications enable a more selective expression or suppression of genes. DNA methylation is a highly studied epigenetic mechanism that plays an important role in regulating gene expression. As noted previously, abnormal DNA methylation can induce related diseases such as stroke [27], cardiovascular disease [28], and malignancies [29]. However, the role of DNA methylation in the pathogenesis of ischemic stroke is yet to be fully elucidated.

In this study, we evaluated the potential association of *AHCY* methylation with ischemic stroke and determined a significantly higher frequency of this epigenetic modification among ischemic stroke patients relative to controls, irrespective of sex and age. *AHCY*, which is located on chromosome 20, contains ten exons and encodes *AHCY*. This broadly expressed enzyme hydrolyzes SAH, which is produced by the demethylation of SAM, a molecule that participates in the methylation of DNA and histones [30]. *AHCY* catalysis produces homocysteine from the metabolic precursor SAH and, therefore, the inhibition of *AHCY* can result in an accumulation of SAH in cells. Additionally, SAH is a potent feedback inhibitor of most SAM-dependent transmethylation reactions, including those related to the methylation of cellular components such as DNA, RNA, lipids, proteins, and neurotransmitters [17,31]. Therefore, *AHCY* methylation may affect *AHCY* expression and activity, leading to disordered SAH metabolism, with negative impacts on the regulation of biological processes. A previous study demonstrated that mutations in *AHCY* were associated with prognosis in neuroblastoma patients [32], suggesting that this gene could be considered as a potential prognostic factor in this context [23]. Giusti et al. identified a potentially significant difference in the allelic frequency of the *AHCY* polymorphism rs819146 between ischemic stroke patients and control subjects according to the recessive model [33]. Furthermore, the *AHCY* haplotype was reported as a susceptibility factor for abdominal aortic aneurysm, and this association is independent of the homocysteine modulatory role of this enzyme [34]. Therefore, *AHCY* methylation may regulate the enzymatic activity of *AHCY*, resulting in increased homocysteine levels in patients with cerebrovascular disease.

Many biomarkers of brain diseases involve DNA methylation. Jakubowski and Labrie [35] reported that DNA methylation may be a novel biomarker with potential etiological relevance in Parkinson’s disease. Thon et al. suggested that the O6-methylguanine-DNA methyltransferase promoter methylation status is crucial in glioblastoma treatment [36]. Moreover, *TRIM59* and *KLF14* hypermethylation in blood cells may be a valuable predictor of the molecular processes underlying familial Alzheimer’s disease [37]. Recent studies suggested that age-related DNA methylation changes were independent predictors of ischemic stroke outcomes [38,39]. Li et al. [40] demonstrated a significant difference in the *AHCY* methylation levels between ischemic stroke patients and controls older than 60 years of age. We similarly demonstrated a significant difference in the *AHCY* methylation levels between ischemic cases and controls, although this result was consistent in all age groups. Our ROC analysis further confirmed that *AHCY* methylation is a significant predictor of the risk of ischemic stroke.

Our study had some limitations of note. First, our analysis of the association between *AHCY* DNA methylation and the risk of ischemic stroke focused on only one portion of the CGI. Therefore, our results may not be applicable to all *AHCY* sequences. Second, we did not analyze the transcription or translation of *AHCY*. Therefore, a mechanistic study of the association between *AHCY* methylation and ischemic stroke is needed. Third, the sample size of this study was small. Further evaluations with larger samples are needed to elucidate the relationship between *AHCY* methylation and ischemic stroke, particularly in various ethnic populations.

## CONCLUSION

Our findings confirm a significant correlation between the *AHCY* DNA methylation level and the risk of ischemic stroke and suggest that *AHCY* methylation may be a useful diagnostic biomarker of ischemic stroke.
